# Availability of Empty Zona Pellucida for Generating Embryonic Chimeras

**DOI:** 10.1371/journal.pone.0123178

**Published:** 2015-04-28

**Authors:** Chi-Hun Park, Young-Hee Jeong, Dong-Kyung Lee, Jae Yeon Hwang, Kyung-Jun Uh, Su-Cheong Yeom, Curie Ahn, Chang-Kyu Lee

**Affiliations:** 1 Institute of Green Bio Science and Technology, Seoul National University, Pyeong Chang, Kangwon do, Korea; 2 College of Agriculture and Life Science, Chonnam National University, Gwangju, Korea; 3 Department of Agricultural Biotechnology, Animal Biotechnology Major, and Research Institute of Agriculture and Life Science, Seoul National University, Seoul, Korea; 4 International Agriculture Technology Graduate School, Seoul National University, Pyeong Chang, Kangwon do, Korea; 5 Division of Nephrology, Seoul National University College of Medicine, Seoul, Korea; USA, UNITED STATES

## Abstract

In the present study we used an empty zona pellucida derived from hatched blastocysts as an alternative source for embryo aggregation and compared results with the conventional microwell method. Denuded 4-cell stage porcine embryos were aggregated by introduction into an empty zona or placement within a concave microwell. The present study showed that although the rate of aggregate formation was similar, the blastocyst rates and allocation of more cells to the inner cell mass (ICM) in the resultant aggregates were increased significantly more in the empty zona than in the microwell. Notably, using an empty zona showed no limitations with regards to the increased number of embryos aggregated or embryonic stages for aggregation, while partial or no aggregation frequently occurred in the microwell. The discrepancy may be due to the difference of microenvironments where the embryos were placed namely, the presence/absence of zona pellucida. We hypothesize the success of the empty zona in generating aggregates is due to the physical aggregation of individual embryos allowing closer contact between the blastomeres and/or embryos compared with a concave microwell. These results indicate that aggregation conditions could influence overall production efficiency and developmental potential of aggregates, suggesting physical restraint via empty zona that provide three-dimensional pressures is an important factor for successful embryo aggregation.

## Introduction

After the first chimeric mice were reported in the early 1960s [1], experimental chimera has served as a powerful research tool for studying cell lineage determination and for determining potency of various stem cells, as well as for generating gene-modified mice [2]. The chimera competency of embryonic stem (ES) cells and induced pluripotent stem (iPS) cells in mice and embryo-embryo aggregation in pigs has recently been utilized for generating specific organs [3,4]. Mouse chimeras can be generated by combining 2 or more dissected or whole embryos [5] and either by direct microinjection of ES cells into a host blastocyst [6] or by co-culture with a host diploid or tetraploid embryo [7]. Blastocyst injection is the most widely used method for producing ES cell chimeric mice. The current processes creating an ES-derived chimera with this technique is of limited use in other species where putative ES or iPS cells are lacking in chimera-forming abilities and in contributing to the germ-line [8].

Because cloned or tetraploid mouse embryos have impaired developmental potentials with a reduced cell number of blastocysts, embryo aggregation has been used as an attempt to compensate for this deficiency [9,10]; increasing the total cells in chimeric blastocysts by aggregating multiple embryos can improve the pregnancy rate and the yield of live-born pups [11,12] which is consistent with observations made in other mammalian species, (e.g. rabbits, horses and pigs) [13]. In addition, pig embryo aggregates have shown increased OCT4 transcription levels and improved *in vitro* development [14,15]. Currently, embryo aggregation is considered to be a practical and reliable method for generating experimental chimera in non-rodent species including pigs and primates [8].

Zona pellucida (ZP) is a specialized extracellular matrix surrounding the plasma membrane of mammalian oocytes having various roles in fertilization and development. During the fertilization process, ZP prevents polyspermic penetration through its physical hardness due to release of cortical granule contents [16]. During preimplantation development, this cover plays a protective role for the embryo from rejection by the maternal immune system and from infectious agents [17]. Several investigations have focused on the absence of ZP and the potential impacts on *in vivo* development [1,18,19]. The ZP may be involved in maintaining the cell arrangement of cleavage stage embryos to ensure subsequent successful cell lineage differentiation [20]. Moreover, the zona-dispersed demiembryos have shown imbalanced ratios of the inner cell mass (ICM) compared with the trophectoderm (TE) and altered expression of Cdx2 and Oct4 [21]. Zona removal (mechanically or by exposure to pronase) induces a transient alteration in DNA methylation status of mouse embryos [22]. Despite potential influences of the ZP dispersion on early embryo development, zona removal is apparently an inevitable step for aggregating embryos.

Embryonic chimeras are commonly generated by co-culture of 2- or 3-embryos within a concave microwell, which is a well-described method; however, this method is still hampered by some technical limitations [23]. For example, embryos frequently fail to aggregate into a single embryo in droplets due to a very minor impact when handled. The aim of the study was to determine the availability of empty zonas an alternative source for embryo aggregation as previously determined in mice [24], and its effects on the aggregation efficiency of porcine parthenogenetic and cloned embryos and cell distribution pattern in the resultant aggregates. During *in vitro* embryo culture, the empty zona derived from a hatched blastocyst is considered as waste and thus is generally discarded at the end of experiments. In this study, the empty zona obtained from the porcine parthenotes on day 7 or 8 of *in vitro* culture was employed for embryo aggregation.

## Materials and Methods

### Ethics statement

All animal studies were performed after receiving approval of the Institutional Animal Care and Use Committee (IACUC) of Seoul National University (IACUC approval No. SNU-140328-2, S1 Fig). Porcine ovaries were provided by the regional slaughterhouse (Pyeongchang, Korea).

### Oocyte maturation

Ovaries were collected from prepubescent gilts at a local slaughterhouse (Pyeongchang Co.) and transported to the laboratory in 0.9% (w/v) NaCl supplemented with 100 mg/mL streptomycin sulfate (Amresco, Solon, OH, USA) within 30 min at 37°C. Only cumulus-oocyte complexes (COCs) were collected and washed twice with DPBS supplemented with 0.01% polyvinyl alcohol). After washing, 40–50 COCs were transferred to 500 μL of an IVM medium (TCM-199; Invitrogen, Carlsbad, CA, USA) supplemented with 10 ng/mL epidermal growth factor (EGF), 1 μg/mL insulin (Sigma-Aldrich Corp., St. Louis, MO, USA), 4 IU/mL eCG (Intervet, Boxmeer, The Netherlands), hCG (Intervet) and 10% (v/v) porcine follicular fluid (pFF) and were cultured for 22 h. Next, the COCs were transferred to an IVM medium without hormones and were cultured for a further 22 h at 38.5°C in an atmosphere containing 5% CO_2_ and 100% humidity.

### Generation of parthenotes and cloned embryos

For generating parthenotes, matured oocytes were activated by an electric pulse (1.0 kV/cm for 60 μsec) in activation medium (280 mM mannitol, 0.01 mM CaCl_2_, 0.05mM MgCl_2_) using a BTX Electro Cell Manipulator (BTX, Holliston, MA, USA), followed by 4 h of incubation in PZM3 medium containing 2 mmol/L 6-dimethylaminopurine.

Adult fibroblast cells were obtained from abdominal skin biopsy of a female Yucatan minipig. The biopsy was performed under isoflurane anesthesia following ketamine (25 mg/kg) and xylazine (0.5 mg/kg) premedication.Growing cells at a density of 3 x 10^5^ cells per well were transfected with 2 μg of linearized pEGFP-N3 or pDsRed2-N1 using Lipofectamine LTXTM Reagent (Invitrogen) as suggested by the manufacturer. At 24 h after transfection, the transfected cells were passaged and selected with 0.3 mg/mL of neomycin G418 (Gibco) for 3 weeks. Somatic cell nuclear transfer (SCNT) was performed as previously described [25]. Briefly, enucleation was conducted in DPBS supplemented with 0.4% bovine serum albumin (BSA) and 5 mg/mL cytochalasin B. Matured oocytes were enucleated by aspiration with an enucleation pipette (Humagen, Charlottesville, VA, USA). After enucleation, GFP- or RFP-positive cells were mechanically collected and introduced into the perivitelline space of an enucleated oocyte. Fusion of injected oocytes was induced in a fusion medium (280 mM mannitol, 0.001 mM CaCl_2_, and 0.05 mM MgCl_2_) by 2 DC pulses (1-sec interval) of 2.0 kV/cm for 30 μsec. After 1 h of incubation, reconstructed oocytes were electrically activated as described above. Post-activated oocytes were cultured in PZM3 for 144 h. Embryo culture conditions were maintained at 38.5°C in an atmosphere containing 5% CO_2_, 5% O_2_ and 100% humidity.

### Aggregation process

**Fig 1:**
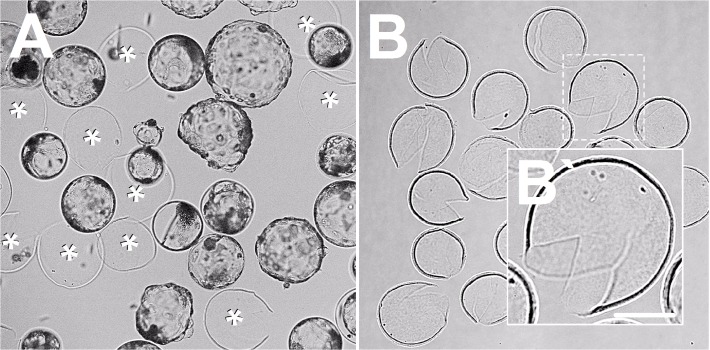
Parthenogenetic blastocysts and zona pellucida (ZP). After electrical activation, the embryos were cultured for 7 to 8 days. Blastocysts and freed ZP (asterisks) with various sizes and shapes were observed. **(A)** The thin, stretched, ruptured, empty zona were observed after shedding. **(B)** The empty zona with a large slit (solid arrowhead) or with a narrow slit (open arrowhead) were discarded. Only empty zona shaped like the video game icon "Pac-Man" with a proper slit size were collected. **(B`)** Bar in B`is 100 μm.

**Fig 2:**
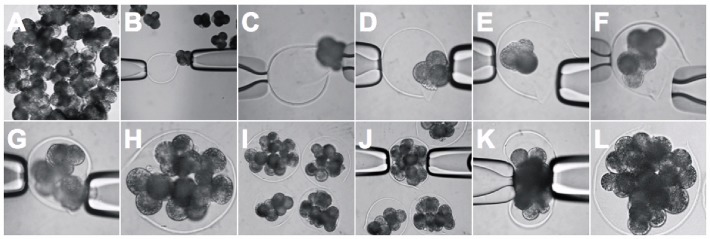
Injection of 4-cell stage embryos into empty zona. After dissolving the zona pellucida (ZP), the denuded parthenotes at the 4-cell stage **(A)** were injected into the empty zona *via* micromanipulation with hollow glass needles **(B-G)**. Multiple embryos with various numbers were reconstructed using the empty zona method **(H and I)**. The embryos injected into an empty zonae were gently pressed around through the glass needle, causing them to bind together more tightly **(J-L)**.

Empty zona pellucida of various sizes (range, 150~300 μm in diameter) were collected (Fig 1) and washed free of any debris in DPBS with 0.2% BSA by gentle pipetting and stored in PZM3 with 0.2% BSA under the same embryo culture condition until use. At the end of the experiments, empty zona were reused in the same manner as described above. At 2 days of culture after electrical activation, the 4-cell stage parthenogenetic embryo ZPs were removed by short exposure to acidic Tyrode’s solution (Gibco). The denuded embryos were introduced into a slit in the empty zona using a micromanipulator with hollow glass needles (Fig 2) or placed into each concave microwell (300 μm in diameter, S2 Fig), created by a smooth depression using darning needles, as described previously [14].

### Differential staining and cell number counts

For examining the blastocyst cell counts, the numbers of ICM and total cells of individual aggregates collected on day 7 were counted by differential staining [26] using a fluorescence microscope.

### Statistical analysis

The data obtained in this study were analyzed using the GraphPad Prism statistical program (GraphPad Software, San Diego, CA, USA). Data on *in vitro* developmental and aggregate formation rates were analyzed using analysis of variance (ANOVA) and Dunnett’s test. Comparisons between the 2 groups were performed using Student's *t*-tests. All data were expressed as mean values ± standard error of the mean (SEM). A probability of *p* < 0.05 was considered to indicate statistical significance.

## Results

### Empty zona collection after shedding of blastocysts

As shown in Fig 1A, various empty zona sizes and shapes were obtained from late blastocysts on day 7 or 8 of *in vitro* culture. In this study, we did not use the empty zona from the embryos of *in vitro* fertilization to exclude the possibility of detrimental effects due to many dead sperm surrounding the ZP. The empty zona with relatively large slits (larger than twice the embryo's diameter) created during hatching were discarded because the aggregates tended to leak out through the large slit breaks (Fig 1B, solid arrowhead). Additionally, the empty zona with too narrow slits making injection into the denuded embryos difficult and in turn, possibly injuring their membranes during the injecting process were not used (Fig 1B, open arrowhead). Therefore, we used primarily the empty zona were similar to that in Fig 1B`.

### Introduction embryos into an empty zona

For this experiment, we used parthenogenetic embryos which can be easily generated and minimized possible artifacts from other embryo types; *in vitro* fertilized embryos that show high incidence of aneuploidy caused by polyspermic penetration or mixed sexes and cloned embryos with poor development and labor-intensive process. The zona-removed 4-cell stage embryos were introduced into a slit in the empty zona using a micromanipulator (Fig 2). After injection, the embryos were gently pressed and squeezed around by rotating through hollow glass needles (Fig 2H–2L), causing them to lie in closer contact. This step may not be critical, but produces a more efficient outcome in which embryos are weakly attached. Thus, partial aggregation that frequently occurs in a droplet could be reduced when this step was added (S1 Table). Once embryos are placed in an empty zona, they remain in contact even if rough handling or repeat pipetting occurs. The conventional aggregation was also conducted using the microwell method (S2 Fig).

### Microenvironments and their impact on aggregation and *in vitro* development of aggregates

**Fig 3:**
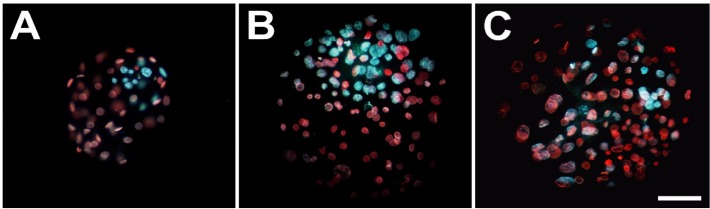
Representative images of differentially-stained porcine day 6 blastocysts. A singleton **(A)** and 3X aggregate blastocyst with compact **(B)** or scattered or non-compact **(C)** inner cell mass (ICM). Using a conventional differential staining method, ICM were labeled with Hoechst 33342 (blue) and trophectoderm (TE) nuclei with propidium iodide (red); photographed under an epifluorescent microscope. Scale bars: 100 μm.

To determine the applicability for generating embryonic chimeras, we employed the empty zona method (n = 33) and the conventional microwell method (n = 35) to generate aggregates with multiple embryos (3X). After culturing for 48 h, although a few aggregates (n = 3) were obtained with a small number of blastomeres arrested, most (n = 32) underwent the well-formed compacted morula stage (full aggregation) when using the empty zona with an average size of 200 μm, progressing to the blastocyst stage (Table 1). In the microwell method, not only full aggregation (n = 28), but also incomplete aggregates with separated blastocoel cavities (partial aggregation) and no aggregation (n = 7; S2 Fig) were observed. The aggregates that failed to aggregate into a single blastocyst were excluded from further analyses. The overall aggregate formation was slightly lower in the microwell (81.3%) than in the empty zona method (96.0%), but was not significantly different (*p* > 0.05). However, the rate of blastocyst formation was significantly higher in aggregates derived from the empty zona method (92.7%, *p* < 0.0001) than from the microwell (68.4%, *p* < 0.05) and the control singletons derived from the denuded embryos (45.5%). At the end of culture (72 h), the number of total and ICM cells was determined using a differential staining technique (Fig 3). The mean number of total cells (32.8, *p* < 0.0001) was less in singletons than aggregated blastocysts in aggregates of the microwell (80.1) and empty zona (85.7) methods. However, the resultant blastocysts had significantly greater number of ICM cells and a higher ICM/total cell ratio in the EZ method (25.7 and 3.0, respectively; *p* < 0.0001) compared with the microwell method (14.3 and 5.2) and the control (6.8 and 6.1). This may be a result of many aggregates exhibiting a non-compacted ICM in the blastocysts derived from the microwell method (S3 Fig), while such anomaly was rarely observed in aggregates produced by the empty zona method. The physical trapping by ZP can apparently impact aggregation formation of embryos and cell allocation in the resultant aggregates.

**Table 1:** Developmental rate of triple-aggregated embryos produced using the EZ and conventional microwell methods.

Groups	Number examined	Number (%)aggregated		Number (%)blastocyst		Cell number		
						Total (n)	ICM	Ratio
Control^†^	91	45^‡^	(49.3 ± 2.1)	42	(45.5 ± 2.2)^a^	32.8 (12)^a^	6.8^a^	6.1^a^
Microwell	35	28	(81.3 ± 3.7)	24	(68.4 ± 2.4)^b^	80.1 (9)^b^	14.3^b^	5.2^a^
empty zona	33	32	(96.0 ± 1.8)	31	(92.7 ± 2.0)^c^	85.7 (10)^b^	25.7^c^	3.0^b^

The rates of aggregation and blastocyst formation were calculated in the embryo on day 5 at the morula stage and on day 7 at the blastocyst stage, respectively. The aggregates with only 1 or 2 blastomeres were also regarded as successful aggregation.

ICM, inner cell mass

^†^ denuded 4-cell embryos were individually cultured in microwells and developed to the blastocyst stage

^‡^ the mean value is the proportions of embryos that became morulae

Each group had 3 replicates. Values are expressed as mean ± standard error of the mean (SEM).

Within the same column, values with different letters (a, b, and c) in superscript are significantly different (*p* < 0.05).

### Effect of increasing the number of embryos on aggregate formation

**Fig 4:**
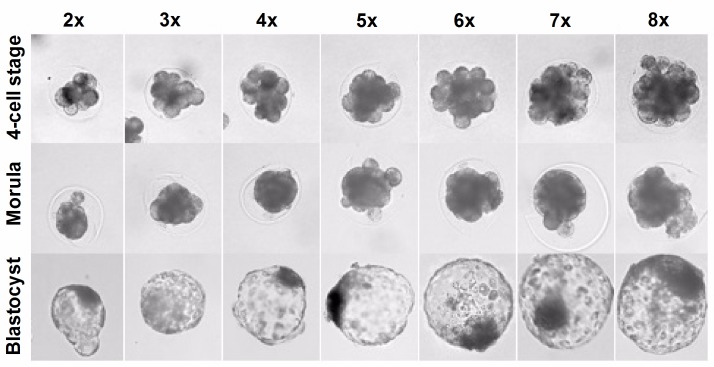
Aggregate formation and *in vitro* development of embryos using the empty zona method. Chimeric morulae and blastocysts derived from 2 (2X), 3 (3X), 4 (4X), 5 (5X), 6 (6X), 7 (7X) and 8 (8X) aggregated embryos were photographed using an optical microscope. The embryos began to compact on day 2 of culturing after introduction and the majority developed to the blastocyst stage.

We tested the ability of empty zona to support aggregate formation and its developmental potential therefore, we examined the 4-cell stage embryo aggregates with various number of embryos in both methods. For this experiment, the denuded embryos were introduced into empty zona of various sizes (the ranges from 150 to 300 μm), depending on the number of embryos used for aggregation (from 2 to 8 embryos) and then cultured individually. A majority of the embryos assembled into a single aggregate *via* our method and progressed to the blastocyst stage (Fig 4). Of the embryos, only 5.3% exhibited partial aggregates but no failed aggregation was observed (data not shown). The aggregation formation (Table 2) did not differ from an increase in the number of embryos examined (75.0% for 2X, 91.7% for 3X, 83.4% for 4X, 91.7% for 5X, 79.2% for 6X, 91.7% for 7X and 91.7% for 8X). Similarly, the blastocyst formation rate among the groups was not significantly different (*p* > 0.05) in the current experimental setting. There was a slight increase in total cells in the resultant blastocysts, but did not translate into a proportional increase in the number of embryos. In contrast, the conventionally-derived aggregates were limited in the number of embryos that became well-formed blastocysts. Notably, most of the aggregates with 5 or more embryos (87%) did not form a single compact aggregate to be counted as full aggregation (S2 Fig). These results indicate the empty zona method may provide a stable microenvironment for robust aggregate formation of embryos with no limitation on the number of embryos employed.

**Table 2:** Aggregate formation and developmental potentials of aggregates with various embryo numbers using the empty zona method.

Groups	Number examined	Number (%) aggregated		Number (%) blastocyst		Total cell number^†^
1X	21	11^‡^	(53.8 ± 7.5)	8	(70.8 ± 10.5)	39.8 * (n = 8)
2X	11	8	(75.0 ± 8.3)	7	(87.5 ± 12.5)	67.9 (n = 7)
3X	13	12	(91.7 ± 8.3)	11	(93.8 ± 6.3)	86.0 (n = 10)
4X	11	9	(83.4 ± 9.6)	8	(87.5 ± 12.5)	93.7 (n = 8)
5X	12	11	(91.7 ± 8.3)	9	(85.4 ± 8.6)	98.6 (n = 9)
6X	10	8	(79.2 ± 12.5)	8	(100 ± 0)	104.8 (n = 8)
7X	9	8	(91.7 ± 8.3)	8	(100 ± 0)	114.3 (n = 8)
8X	12	11	(91.7 ± 8.3)	10	(91.7 ± 8.3)	115.5 (n = 10)

^†^ In the experiment of total cell number, the aggregated morula with the dissociated blastomeres (3 or 4) were downcounted as less as 1X (as represented in Fig 4; 5X and 8X were counted as 4X and 7X, respectively)

1X group is the denuded 4-cell stage embryos that were individually injected into the empty zona

^‡^ the mean value is the proportions of embryo that became morulae

Within the same column, values with different asterisks are significantly different (* *p* < 0.05). Each group has 4 replicates. Values are expressed as mean ± standard error of the mean (SEM).

### Cell allocation of cloned embryo aggregates expressing green fluorescent proteins (GFP) or red fluorescent proteins (RFP)

We verified the methodology could be applied to generate multi-transgenic animals. Using a fetal fibroblast cell strain, the 2 sublines that express GFP and RFP were established and used as nuclear donors for SCNT [25]. Table 3 shows the results of *in vitro* development of the transgenic embryos reconstructed using the GFP and RFP. The embryos expressing GFP (*p* < 0.05) had significantly higher rates of cleavage (70.0 ± 6.5) and blastocyst formation (31.8 ± 2.8) than the RFP-expressing embryos (50.9 ± 5.2 and 16.2 ± 0.8, respectively). In total cell numbers, there was no significant difference between the GFP (38.3 ± 3.5) and RFP (32.6 ± 3.5) cloned blastocysts (*p* > 0.05).

**Table 3:** Aggregate formation and *in vitro* development of cloned embryos expressing GFP and RFP.

Groups	Number examined	Number (%) cleaved		Number (%) aggregated		Number (%) blastocyst		Number oftotal cells
GFP	188	132	(70.0 ± 6.5)^a^			59	(31.8 ± 2.8)^a^	38.9 ± 3.8^a^ (n = 20)
RFP	187	94	(50.9 ± 5.2)^b^			30	(16.2 ± 0.8)^b^	32.7 ± 3.5^a^ (n = 18)
GFP^(4X)^	32			25	(77.9 ± 2.4)^a^	19	(76.0 ± 6.2)^c^	91.0 ± 5.1^b^ (n = 11)
RFP^(4X)^	31			16	(57.2 ± 4.7)^b^	8	(46.7 ± 6.2)^a^	71.1 ± 6.4^b^ (n = 8)
GFP/RFP^(4X)^	24			17	(70.3 ± 4.0)^a^	12	(67.7 ± 5.1)^c^	84.4 ± 3.1^b^ (n = 10)

GFP, green fluorescent protein; RFP, red fluorescent protein

The cleavage rate was recorded 42–44 h post activation. All percentage data expressed are mean values ± standard error of the mean (SEM). Different letters (a, b, and c) in superscript indicate significant differences in mean values (*p* < 0.05). Data on blastocyst rate and total cells of RFP and aggregate groups was compared to the GFP group using one-way analysis of variance (ANOVA).

**Fig 5:**
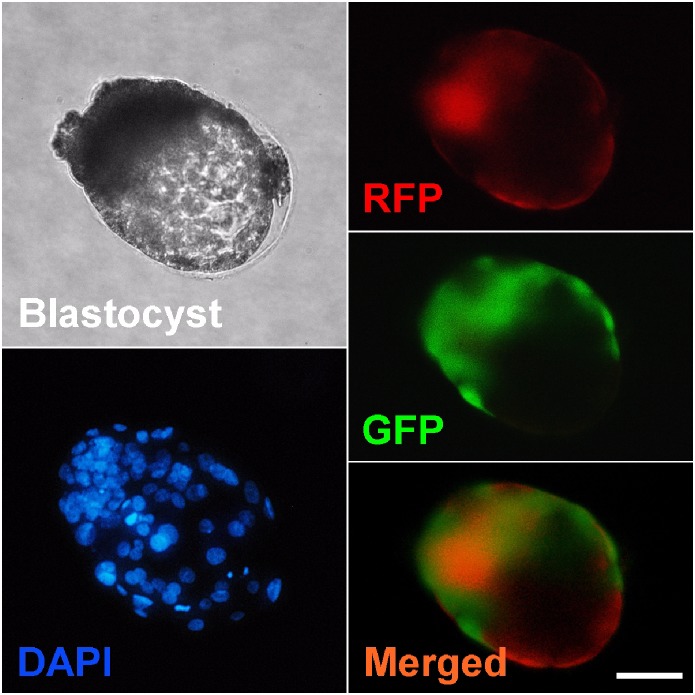
Green fluorescent protein (GFP) and red fluorescent protein (RFP) expression in 4X cloned aggregates using the empty zona method. Two cloned embryos derived from the GFP or RFP cell line were aggregated using the empty zona method. A cloned aggregate (4X) was photographed **(A)** and the cell distribution pattern traced using a fluorescent microscope.

Based on this finding, 4-cell stage GFP and/or RFP embryos were aggregated *via* the empty zona method. The comparison clearly shows the aggregation process could lead to an approximate 2-fold increase in overall blastocyst rates and total cell number of aggregates, compared with their intact counterparts (*p* < 0.05). However, a significant difference was observed between GFP^(4X)^ and RFP^(4X)^ groups in aggregate formation and blastocyst rate. This trend was not detected when equal numbers of GFP/RFP^(4X)^ were intermixed. In addition, to evaluate the extent of intermingling of embryonic cells in aggregates, the resultant aggregates with GFP/RFP embryos were imaged using a fluorescence microscopy to trace the cell distribution pattern in aggregates. The results showed all aggregates (4X) with intermingled GFP/RFP-positive cells (Fig 5). In particular, GFP-positive cells tended to contribute to the ICM more than to the TE, while the cells expressing RFP broadly dispersed to both layers. However, there was no apparent tendency to allocate exclusively to the ICM or TE. The embryonic cells possessing a high potential for development are apparently more likely to contribute to the embryonic part, but not exclusively. Thus, these results may indicate that developmental capacity of component embryos in aggregates affects the overall aggregation efficiency and cell distribution pattern within resultant aggregates.

## Discussion

Our data showed an increased blastocyst rate and number of total cells in aggregates than singleton, supporting the notion that embryo-to-embryo aggregation may rescue developmentally impaired embryos in aggregates [11,12]. Moreover, the blastocyst formation and the ICM cell number were significantly higher in the empty zona method than in the microwell method, whereas the total cell number in resultant aggregates was similar between the two methods. The only difference between the methods was the presence/absence of ZP, indicating its presence caused this difference. Although ZP is not fundamental for *in vitro* development of early mouse embryos to the blastocyst stage, many studies have shown the possible impacts on zona removal in developing embryos. Notably, zona absence could lead to disrupted regulation of polarity and incorrect division patterns of early stage embryos. In pig embryos, the zona removal adversely affected blastocyst formation and zona-free sheep cloned embryos frequently exhibited a significant decrease in ICM cell number and the OCT4 transcripts at the blastocyst stage [27]. Note that, using free-zonae derived from blastocysts has already been tested in earlier studies on the effect of removal of the zona pellucida on subsequent development of blastocysts, but has only been done with half-ablated embryos or single blastomeres [28–30]. Given that the bisected embryos or single blastomeres cannot fill in the empty space of a host empty zona, even those with the zona-confined had weak interactions between blastomeres and abnormal growth patterns, which occurred in zona-dispersed counterparts [18]. The present study does not address directly the issue of whether zona removal has negative effects on early developing porcine embryos, but previous literature implicates ZP as an important component in some aspects of early embryonic development. These observations with the previous studies indicating indicate that ZP may have contributed to changes in cell fate and developmental potential of early pig embryos [30,31].

The present results showed that using a microwell led to progressive increases in the rate of failed aggregation with increasing number of embryos aggregated. The zona-free embryos were easily separated from one another and frequently underwent the loss of blastomeres before the compacted morula stage [27], so it would seem that a simple coculture system using a microwell can actually create a deleterious microenvironment in denuded embryos. By contrast, the empty ZP in which the denuded embryos are restrained keeps them firmly attached together during the aggregation process thereby enabling them to fully aggregate. The empty zona method showed no limitation for aggregation regarding the number of embryos (8 or even higher) while the efficiency was diminished in the microwell method if the number of embryos (more than 4) was increased. We hypothesize the increase in cell-to-cell contact between embryos enhances aggregational capacity of the developing embryo. Thus, these results indicate the possible impacts that currently exist in conventional aggregation methods in relation with zona removal can be reduced using the empty zona method. Accordingly, using an empty zona with the proper size is important primarily to consider for embryo aggregation and may lie in the number of embryos used, allowing them to fill tightly in place. Together, empty zona provide a conducive and stable environment to ensure physical restraint of embryos, allowing for successful aggregate formation and preimplantation development. Apart from its availability, the present results also showed the total cells in the resultant blastocysts did not increase proportionally as increased number of embryos aggregated (more than 3). These results are in agreement with previous studies suggesting a possible mechanism of size regulation by controlling cell proliferation activity in aggregates [32]. Therefore, although aggregates with 4 or more embryos did not severely compromise their development *in vitro*, there may be no developmental advantage using more than 3 embryos.

Our results also showed that aggregation of cloned embryos enhanced blastocyst formation compared with intact cloned embryos, but differences in aggregate formation and blastocyst rate between embryos cloned from different donor cells were observed. In the present study, the varying developmental potential of cloned embryos resulted in reduced ability of embryos to aggregate, similar to a previous study [33]. Thus, these results indicate the combination of multiple embryos with different developmental potentials affects the overall aggregation efficiency and cell distribution pattern within resultant aggregates.

The impact of the embryonic stage on embryo aggregation and the optimal timing may vary for different species. Our previous observations showed the 4-cell stage is the optimal stage for aggregation of porcine embryos [14]. In preliminary experiments, we observed no limitation regarding the embryonic stages (1- to 8-cell stage) in aggregation formation *via* the empty zona method (data not shown) indicating that using empty zona allows flexibility in choosing developmental stages of embryos for aggregation. Accordingly, after introduction into an empty zona, the embryo can immediately be transferred into a recipient without requiring additional culture periods until becoming a single aggregate [34]. This is a significant advancement because generating live pig chimeras from transferred blastocysts is technically challenging since the *in vitro* culture systems of porcine embryos are still considered suboptimal [35].

Herein, we determined the availability of empty zona as an alternative source for embryo-embryo aggregation. A novel technique for embryo aggregation, the empty zona method, provides a physical space using a natural component, which allows a greater opportunity for physical contact between embryos for successful aggregation to occur. Additionally, it allows safe and easy handling of embryo during *in vitro* experiments, while the denuded embryo tends to become sticky and fragile. Based on the present findings, the empty zona method can generate embryonic chimeras with greater efficiency than the conventional microwell method and can be applied to practical chimera production systems for other mammalian species. However, further confirmation by *in vivo* studies is needed to validate *in vitro* results of this method.

## Supporting Information

S1 Table Aggregation efficiency of triple embryos produced using the conventional microwell method with or without pressing around. (DOCX)

S1 Fig Approval of the Institutional Animal Care and Use Committee (IACUC). (PDF)

S2 Fig The microwell aggregation of 4-cell stage embryos. The microwell aggregation was conducted by placing the denuded embryos in a smooth depression using darning needles. Two, three, four and five aggregates are shown.
(TIFF)

S3 Fig Representative images of differentially-stained porcine day 6 blastocysts. Inner cell mass (ICM) cells are shown in blue and TE cells are shown in red. Porcine day 6 chimeric blastocysts with well formed (A) a few, scattered (B) and disaggregated (C) ICM. Scale bars: 100 μm.
(TIF)
