# Supplementary material for: Availability of Empty Zona Pellucida for Generating Embryonic Chimeras
Source: PLoS One. 2015 Apr 28;10(4):e0123178. doi: 10.1371/journal.pone.0123178 (PMC4412630; doi:10.1371/journal.pone.0123178)
Supplement: S1 Table — (DOCX) [file pone.0123178.s001.docx]

|  | | | | | |
| --- | --- | --- | --- | --- | --- |
| Gently pressing^§^ | Number aggregated^†^ | Number (%) partly | | Number (%) well-formed | |
| + | 17 | 2 | (10.7 ± 6.9) | 15 | (89.3 ± 6.9) |
| - | 18 | 6 | (31.3 ± 10.9) | 12 | (68.7 ± 10.9) |
| ^§^ triple embryos injected into an empty zona that were gently pressed, then removed from the empty zona by aspiration. ^†^ the rate of aggregation was calculated in the embryo on day 5 at the morula stage. Each group had 5 replicates. Values are expressed as mean ± standard error of the mean (SEM). | | | | | |
