# Supplementary material for: Availability of Empty Zona Pellucida for Generating Embryonic Chimeras
Source: PLoS One. 2015 Apr 28;10(4):e0123178. doi: 10.1371/journal.pone.0123178 (PMC4412630; doi:10.1371/journal.pone.0123178)
Supplement: S1 Fig — (PDF) [file pone.0123178.s002.pdf]

Seoul National University

Institutional Animal Care and Use Committee

599 Gwanak-ro, Gwanak-gu, Seoul 151-742

Tel :82-2-880-5152 FAX:82-2-873-0002

[Date]
September 11, 2014

Principle Investigator. Lee, ChangKyu
(College of Agriculture and Life Sciences, Seoul National University)

SNUIACUC Approval No. : SNU- 140328-2
Title of proposal : Gene expression analysis in various organ and adudlt fibroblast cell line derivation in pig
IACUC Approval Date : March 31, 2014

To whom it may concern..

This letter is to officially notify you that the IACUC on Seoul National University has reviewed and approved the above referenced protocol to animal experiments associated with OOOOO.

If you have any questions, please contact SNUIACUC at +82-2-880-5152.

Sincerely,

Chairman
Chair, SNUIACUC
