# Supplementary figures and images for: Availability of Empty Zona Pellucida for Generating Embryonic Chimeras
Source: PLoS One. 2015 Apr 28;10(4):e0123178. doi: 10.1371/journal.pone.0123178 (PMC4412630; doi:10.1371/journal.pone.0123178)

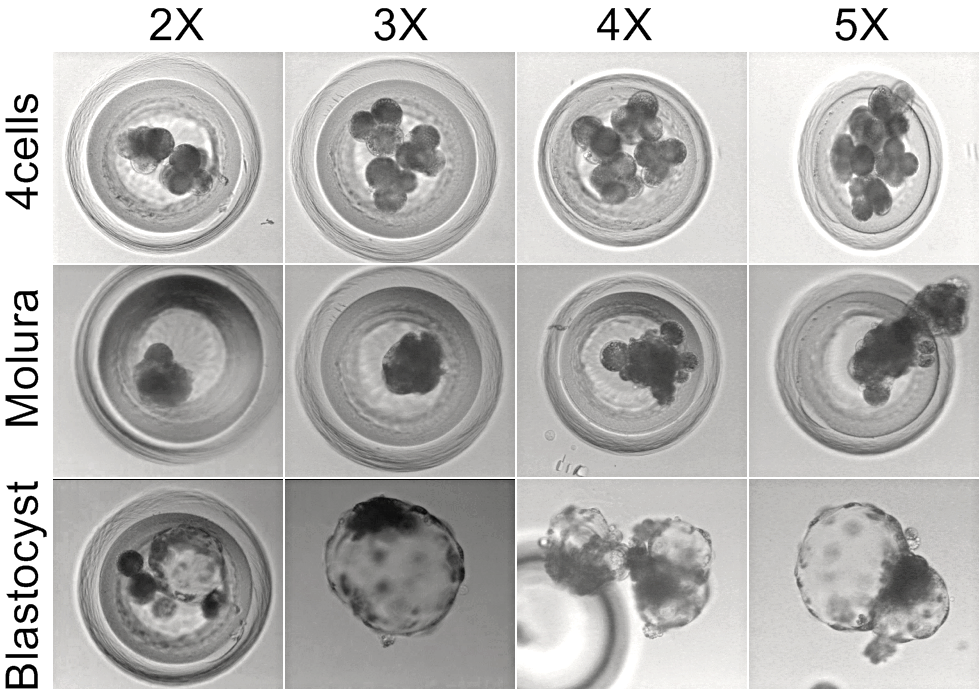

Supplement: S2 Fig — The microwell aggregation was conducted by placing the denuded embryos in a smooth depression using darning needles. Two, three, four and five aggregates are shown. (TIFF) [file pone.0123178.s003.tiff]

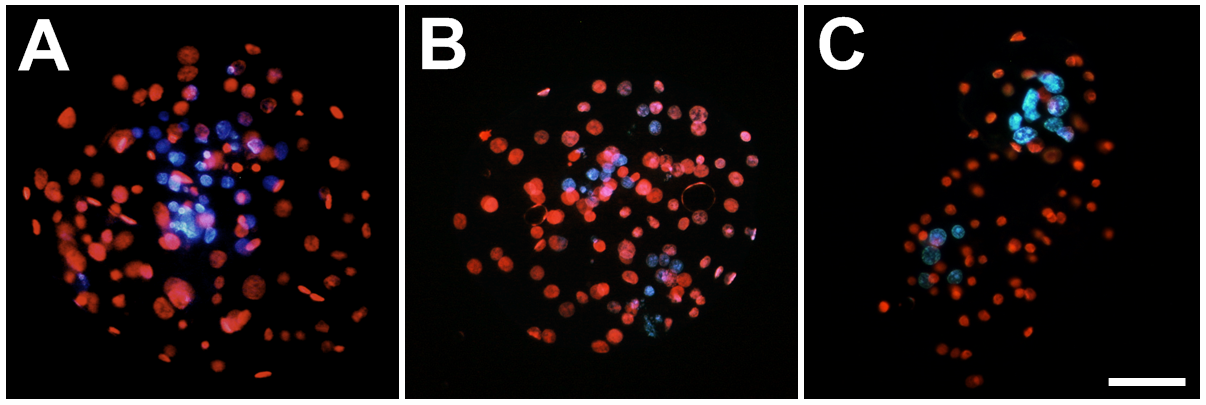

Supplement: S3 Fig — Inner cell mass (ICM) cells are shown in blue and TE cells are shown in red. Porcine day 6 chimeric blastocysts with well formed (A) a few, scattered (B) and disaggregated (C) ICM. Scale bars: 100 μm. (TIF) [file pone.0123178.s004.tif]
